# The role of PARP in DNA repair and its therapeutic exploitation

**DOI:** 10.1038/bjc.2011.382

**Published:** 2011-10-11

**Authors:** M Javle, N J Curtin

**Affiliations:** 1 UT-MD Anderson Cancer Centre, Department of Gastrointestinal Medical Oncology, 1515 Holcombe Boulevard, Unit 426, Houston, TX 77030, USA; 2 Newcastle University, Northern Institute for Cancer Research, Medical School, Newcastle upon Tyne, NE2 4HH, UK

**Keywords:** Poly (ADP-ribose) polymerases, BRCA1, BRCA2, DNA repair, homologous recombination

## Abstract

Historically, PARP inhibitors (PARPi) were developed to potentiate the cytotoxic effect of certain chemotherapeutic agents and are currently being investigated in combination with chemotherapy in diverse cancer types. These agents are also radiosensitisers and clinical trials of PARPi with concurrent radiation are required. It has long been recognised that defective DNA repair pathways lead to tumour susceptibility. Recent studies indicate that tumour cells with defective homologous recombination (HR) repair pathways, the classic example being BRCA mutations, are exquisitely sensitive to PARPi. Defects in HR are not restricted to BRCA-associated tumours and other cancer types may be enriched for HR defects and hence susceptible to PARP inhibition. The identification of predictive markers for sensitivity to PARP inhibition is a priority area for research.

## DNA repair and PARP

Inhibition of DNA repair in cancer cells represents an attractive strategy for potentiating the cytotoxic effects of chemotherapy and radiation and therefore this has been a subject of scientific research for several decades. Of the known DNA repair inhibitors, Poly (ADP-ribose) polymerase inhibitors (PARPi) are furthest along in development and appear promising in a variety of cancer types, including breast and ovarian cancers. The first PARP enzyme was discovered over 40 years ago and PARP-1 is the most abundant and best-characterised member of the family of PARP enzymes (Chambon *et al*, 1963; Sugimura and Miwa, 1994). PARP-1 has a key role in the repair of single-strand breaks (SSBs), resulting from oxidative stress via the base excision repair/SSB repair (BER/SSBR) pathway.

**Figure 1:**
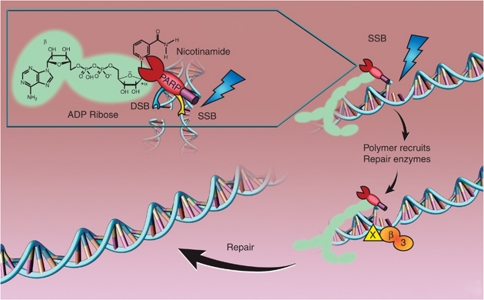
Catalytic activity of PARP-1 and role in DNA BER/SSBR. PARP cleaves NAD^+^ releasing nicotinamide; the ADP-ribose polymers are covalently attached to acceptor proteins, such as PARP itself and histones. These loosen the chromatin and recruit the scaffold protein XRCC1 (X) and other histone remodelling enzymes, which in turn recruits DNA polymerase *β* (*β*) and ligase III (3) to fill in and re-seal the gap. The polymers are degraded by poly ADP-ribose glycohydrolase (PARG), releasing unmodified PARP to bind other DNA breaks.

PARP-1 consists of three conserved, major domains, a NH2-terminal DNA-damage sensing and binding domain containing three zinc fingers, an automodification domain, and a C-terminal catalytic domain. Zinc finger 2 has the strongest affinity for DNA breaks while zinc finger 1 is responsible for DNA-dependent PARP-1 activation, in which zinc finger 3 also participates (Eustermann *et al*, 2011; Langelier *et al*, 2011). PARP-1 is activated by DNA breaks and cleaves nicotinamide adenine dinucleotide (NAD^+^) generating nicotinamide and ADP-ribose (Figure 1). Successive addition of ADP-ribose units to form long and branched chains of poly (ADP-ribose) (PAR), covalently attached to acceptor proteins, including PARP-1, histone and other DNA repair proteins, resulting in polymers adjacent to the DNA breaks. These highly negatively charged polymers form a scaffold and recruit other proteins that are critical in BER/SSBR, for example, XRCC1 (de Murcia *et al*, 1994; El-Khamisy *et al*, 2003). Moreover, other proteins involved in chromatin remodelling, chromosomal organisation, DNA repair and transcription and cell-cycle regulation may also bind to the polymers non-covalently (Gagne *et al*, 2008). PARP-2 was discovered serendipitously when it was noted that cells from PARP-1 knockout mice generate ADP-ribose polymers from NAD^+^ in response to DNA damage. Since the discovery of PARP-1 and PARP-2, a family of 17 proteins with structural similarity to PARP-1 catalytic domain have been identified but only PARP-3, Vault PARP and Tankyrases 1 and 2 have proven ADP-ribose polymerising activity (Schreiber *et al*, 2006). Historically, only PARP-1 and 2 were thought to be activated by DNA damage and the target for intervention but recently PARP-3 has also been implicated in DNA DSB repair (Boehler *et al*, 2011). In this review, we will address the role of PARPi in oncology. Other non-oncological roles of PARPi including neuroprotection, reduction of reperfusion injury and inflammation have been discussed elsewhere (Jagtap and Szabo, 2005).

## Rationale for PARP inhibition in oncology

PARPi are an area of active clinical investigation in oncology as they (1) exploit synthetic lethality in tumours with defective homologous recombination (HR) and (2) potentiate the cytotoxic effect of chemotherapy and radiation.

PARPi were designed to block the catalytic activity of the enzyme and have structural resemblance to the by-product, nicotinamide. One of the earliest inhibitors was the nicotinamide analogue, 3-aminobenzamide (3-AB), which has been extensively used to study PARP inhibition and its effects on chemotherapy and radiation (Bernges and Zeller, 1996; Jacob *et al*, 2007). Further development using ‘analogue by catalogue’, conventional structure–activity relationships and crystal-based drug design reveal that PARPi potency is associated with the carboxamide group in the anti-configuration with respect to the benzamide ring. At the current time, nine PARPi are in clinical development (Table 1). Unlike the other PARPi, which compete with NAD^+^ for the PARP catalytic site, 4-iodo-3-nitobenzamide (iniparib, BSI-201) is reported to eject the zinc ion from the zinc fingers, thereby preventing PARP-1 activation by DNA breaks (Mendeleyev *et al*, 1995). This inhibitor is discussed further below and may also have other ‘off-target’ effects, including inhibition of GAPDH (Bauer *et al*, 2002).

**Figure 2:**
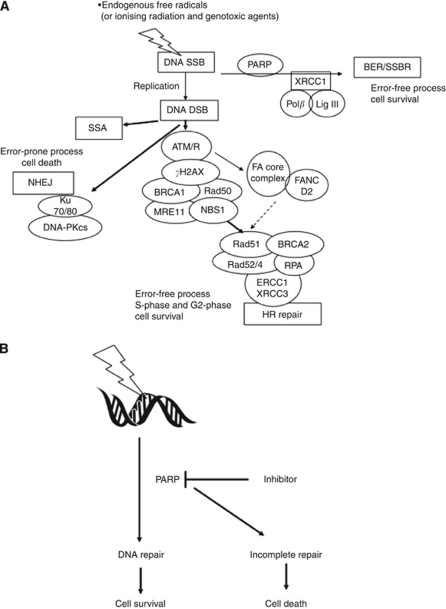
DNA repair and the role of PARP-1/2 in synthetic lethality and chemo- or radiosensitisation. (**A**) Synthetic lethality: endogenously induced, or cytotoxic agent-induced, DNA SSBs are repaired by PARP-dependent BER/SSBR to promote survival. If repair is incomplete, then in proliferating cells, the SSBs will cause replication fork stalling and replication-associated DSBs. These are preferentially repaired by error-free HR to promote cell survival. HR is a complex process involving a multitude of proteins, including BRCA1 and 2, only a few of which are illustrated here. When HR is defective, DSBs persist or are repaired by error-prone SSA or NHEJ, resulting in cell death. (**B**) Chemo- and radiosensitisation. Genotoxic agent-induced DNA breaks normally repaired by PARP-dependent pathways accumulate in the presence of a PARPi, overwhelming alternative repair pathways, converting repairable to unrepairable damage. This ultimately results in cell death.

Synthetic lethality is a term to describe the combined lethal effect of two genetic variations that are otherwise non-lethal when occurring in isolation. In 2005, two groups independently showed the efficacy of PARPi in HR-defective cell lines and tumour xenografts or allografts. Bryant *et al* (2005) noted the profound cytotoxicity of low concentrations of the PARPis, NU1025 and AG14361, in HR-defective cells (BRCA2-deficient V-C8 cells, XRCC3-deficient irs 1SF cells and human breast cancer cells treated with BRCA2 siRNA), while Farmer *et al* (2005) showed that BRCA1 or 2 deficient cells were extremely sensitive to the PARPi KU0058684 and KU0058948 as compared with heterozygous or wild-type cells. Homologous recombination is the principal error-free DNA double-strand break (DSB) repair mechanism and is frequently defective in tumours (Kennedy and D’Andrea, 2006). The synthetic lethality of PARPi in HR-defective cells is generally thought to be due to a failure to repair endogenously generated DNA SSBs in the presence of a PARPi. Such SSB will result in collapsed replication forks and replication-associated DSBs that require HR for repair. In the absence of HR, these lesions prove lethal either because they persist or they can only be repaired by alternative, error-prone pathways including non-homologous end joining (NHEJ) and single-strand annealing (SSA), resulting in genomic instability (Figure 2A). Indeed, recent data suggest that in an HR-defective background, PARP inhibition promotes error-prone NHEJ and that an intact NHEJ and 53BP1 signalling pathway is needed for synthetic lethality (Bunting *et al*, 2010; Patel *et al*, 2011). BRCA1 and BRCA2 are important components of the HR pathway and patients harbouring mutations in these genes have an increased risk of breast, ovarian, prostate and pancreatic cancers. Carriers of BRCA1/2 mutations have one functional allele, and can therefore conduct HR repair in normal tissues, but tumour development is dependent on somatic inactivation of the second allele rendering them defective in HR (Welcsh and King, 2001). Thus, PARPi should only kill the HR-defective tumour cells and not the normal host tissues. These exciting results have spurred research worldwide in this area and several clinical trials are currently ongoing in diverse tumour types (Table 1).

## Chemopotentiation and radiopotentiation *in vitro* and *in vivo*

PARPi were originally investigated as chemo- and radiosensitising agents, before their development in the BRCA-deficient cancers. This approach has been used to augment the cytotoxic effects of the DNA methylating agents (e.g., temolozolomide (TMZ)), topoisomerase-1 inhibitors (e.g., irinotecan and topotecan) and ionising radiation (Curtin, 2005). A large body of evidence, accumulated over the last 20 years, indicates that the mechanism is via inhibiting the PARP-mediated repair of DNA breaks induced by these agents (Figure 2B).

Temolozolomide has limited clinical utility other than for neurological malignancies and melanoma. Addition of PARPis may change this paradigm. Impressive *in vivo* anti-tumour effect was noted when TMZ was combined with PARPi in diverse tumour types including B-cell lymphoma, colorectal, lung, pancreatic, ovarian, breast and prostate cancers (Calabrese *et al*, 2004; Tentori and Graziani, 2005; Donawho *et al*, 2007). Defects in mismatch repair (MMR) are associated with TMZ resistance, but PARPi sensitise MMR-defective cells to the anti-tumour effect of TMZ (Wedge *et al*, 1996; Tentori *et al*, 1999, 2006; Curtin *et al*, 2004; Cheng *et al*, 2005; Horton *et al*, 2009; Vilar *et al*, 2011). Furthermore, the PARPi AG014699 potentiated the cytotoxic effects of TMZ and topotecan in preclinical paediatric tumour models; neuroblastoma and medulloblastoma (Daniel *et al*, 2009, 2010). The combination of PARPi with platinum drugs for BRCA-mutated cancers is also based on sound preclinical rationale. Olaparib (AZD2281) increased the sensitivity of platinum analogues in a genetically engineered mouse model of BRCA1-associated breast cancer (Rottenberg *et al*, 2008). Synergistic cytotoxicity of olaparib and cisplatin was also seen against BRCA2-deficient cells but not against BRCA2-proficient control cells (Evers *et al*, 2008).

Other, novel mechanisms for chemopotentiation have been reported with PARPi. AG14361 and AG014699 have both been reported to have vasoactive effects leading to increased tumour perfusion and hence, potentially improved drug delivery and oxygenation (Calabrese *et al*, 2004; Ali *et al*, 2009). A direct effect on the smooth muscle of the blood vessels was demonstrated using preconstricted rat arteries, with AG014699 being a more potent than the common anti-hypertensive drug, hydralazine (Ali *et al*, 2009). These vasoactive effects may account at least partly for the *in vivo* chemo- and radiosensitisation.

PARPi are potent radiosensitisers in several preclinical tumour models, including lung, colorectal, head and neck, glioma, cervix and prostate cancers (Calabrese *et al*, 2004; Chalmers *et al*, 2010; Powell *et al*, 2010). PARP-1- deficient cell lines were four-fold more sensitive to radiation than their PARP-1-proficient counterparts. PARPi are active not only in proliferating cells, particularly S-phase cells, but also have radiosensitising activity in models of potentially lethal damage recovery (PLDR) in quiescent cells. This latter situation mimics the quiescent radioresistant fraction of tumours. Concurrent treatment with radiation and a variety of PARPi inhibited PLDR by >70% (Calabrese *et al*, 2004; Thomas *et al*, 2007). Interestingly, the radiosensitising effect of PARPi is seen under both hypoxic and euoxic conditions (Powell *et al*, 2010). These promising preclinical radiosensitisation data are yet to be tested in the clinical setting.

It is important to note that the concentrations (*in vitro*) and doses (*in vivo*) needed for chemo- and radiosensitisation are substantially lower than those required for single-agent activity in HR-defective cells and tumours. The therapeutic doses of PARPi in combination studies, therefore, are expected to be lower; this has not always been the case with PARPi combination trials and may underlie the toxicities noted with the latter.

## Role of PARP beyond BRCA

The therapeutic potential of single-agent PARPi extends beyond BRCA1/2 mutation carriers. For instance, it has been suggested that PARPi may be synthetically lethal in sporadic cancers that bear somatic mutations or epigenetic silencing in the various components of the HR pathway. Indeed, recent studies show that AG014699 has single-agent activity in cells and xenografts with BRCA1 promoter methylation (Drew *et al*, 2010). Homologous recombination is a complex process with multiple components, for example, ATM, ATR, CHK1, RAD51 and its homologues, the FANC proteins, MRE11/RAD50/NBS1 (MRN). The PARPi KU0058684 and KU0058948 had single-agent activity in cells defective in several of these proteins (McCabe *et al*, 2006). Other proteins, such as EMSY and PTEN are also implicated as they regulate the activity of other components of the pathway (Cousineau and Belmaaza, 2011). *PTEN* is one of the most commonly mutated tumour suppressors in human cancer and its deficiency was associated with an HR defect. The latter was targeted successfully by the PARPi, olaparib (Mendes-Pereira *et al*, 2009). Given the complexity and multiplicity of the components of the pathway and the variety of tumour types affected, in our view, the term ‘BRCA-ness’ is limiting as it tends to be associated with breast and ovarian cancer, whereas the therapeutic scope for the synthetic lethality of PARPi is potentially much wider. Furthermore, with emerging data that even BRCA mutant cells may be resistant to PARPi through a variety of mechanisms that restore HR function (see below) the BRCA phenotype is even less clear and a better term to describe HR dysfunction is needed.

*Resistance mechanisms* PARPi resistance may be acquired due to intragenic BRCA1/2 mutations that restore the transcript's reading frame thus limiting the effect of BRCA mutations (Sakai *et al*, 2008). Moreover, loss of 53BP1 and NHEJ function also reverse sensitivity to PARPi in BRCA mutant preclinical models by restoring HR function (Bouwman *et al*, 2010; Bunting *et al*, 2010; Patel *et al*, 2011). Other reported mechanisms included upregulation of the *ABCB1a/b* genes, which encode P-glycoprotein multidrug resistance drug efflux pumps (Rottenberg *et al*, 2008). Interestingly, the genetically reverted BRCA2-defective tumours also retain sensitivity to 6TG, which is also dependent on HR for repair and is not a substrate for p-glycoprotein (Issaeva *et al*, 2010).

## Clinical trials of PARPi

There are currently nine PARPi undergoing clinical investigation (Table 1), with or without pharmacodynamic (PD) studies. Pharmacodynamic markers to measure the effect of PARP inhibition include PAR formation in tumour tissue and peripheral blood mononuclear cells as well as assessment of *γ*-H2AX foci. The first clinical trial of a PARPi for cancer was initiated in 2003 and was based on the promising preclinical activity of AG014361 and AG014699 in combination with TMZ (Calabrese *et al*, 2004; Thomas *et al*, 2007). This phase I trial involved a phase 0 component where pharmacokinetic (PK) and PD assays were performed following a single dose of PARPi before the combination of PARPi and TMZ. Pharmacokinetic and PD of AG014699, both as a single agent and after treatment with TMZ were evaluated. Inhibition of PARP activity by >50% was the target PARP-inhibitory dose (PID) in this study (Plummer *et al*, 2008). AG014699 was escalated through five dose levels and PARP inhibition was seen at all doses without any serious adverse events; PID was estimated at 12 mg m^–2^ based on 74–97% inhibition of PARP activity in peripheral blood lymphocytes and a >50% PARP inhibition in tumour biopsies post-treatment. All patients treated at PID showed increases in DNA SSBs. Myelosuppression occurred when high doses of TMZ were combined with AG014699; however, this toxicity was alleviated with TMZ dose reduction. The recommended phase II dose was 200 mg m^–2^ of TMZ with 12 mg m^–2^ of AG014699. AG014699 showed linear PK with no interaction with TMZ. Genotyping studies revealed that in the four patients with the variant CYP2D6 G1846A allele (associated with poor metabolism of AG014699), three experienced clinical benefit (Plummer *et al*, 2008). Further research is needed to examine if this genotype can be used as a predictive marker with AG014699. Dose-limiting myelosuppression was also noted in a phase I trial of INO-101 with TMZ (Bedikian *et al*, 2009). Disappointingly, Khan *et al* (2011) combined olaparib with the alkylating agent, dacarbazine in a phase I trial of patients with advanced melanoma but observed no clinical benefit over dacarbazine alone. Myelosuppression was the commonest toxicity and the maximal tolerated dose was 100 mg of olaparib with 600 mg m^–2^ of dacarbazine.

### Single-agent PARPi trials

Kummar *et al* (2009) conducted the first phase 0 trial of veliparib (ABT-888) in patients with advanced malignancies. The primary study end point was target modulation by the PARPi. In this study, PARP activity, measured after a single dose of veliparib was significantly inhibited at 25 and 50 mg. This innovative, proof-of-concept trial design has the potential of accelerating drug development in oncology with limited use of resources.

Subsequent phase I clinical trials have established the safety of single-agent PARPi in the advanced cancer population as well as in BRCA1/2 mutation carriers. Olaparib was escalated in a phase I clinical trial from 10 mg daily for 2 of every 3 weeks to 600 mg twice daily (Fong *et al*, 2009a). Dose-limiting toxicities at the 400-mg twice daily dose were reversible mood alteration and fatigue while 600 mg twice daily was associated with grade 4 thrombocytopenia and grade 3 somnolence. In all, 200 mg twice daily was selected for further study in BRCA1 or 2 mutation carriers, 19 of which had known BRCA-associated cancers, including breast, ovarian and prostate; 63% of these patients experienced clinical benefit. Impressive response durations were noted in patients with ovarian and breast cancer. Olaparib toxicities were < grade 3 in severity and did not increase in the BRCA mutation carriers.

These promising results led to two phase II studies of olaparib in patients with breast or ovarian cancers having BRCA1/2 mutations (Fong *et al*, 2009b; Tutt *et al*, 2010). The primary study end point for both studies was objective response rate and was higher in the 400-mg arm than the 100-mg arm (41% *vs* 22% in the breast cancer study and 33% *vs* 13% in the ovarian study). Progression-free survival also favoured the higher dose arm. Responses occurred in both BRCA1/2 mutation cases irrespective of race. Treatment was tolerable at both the dose levels; most toxicities were grade 1 or 2 including fatigue and nausea. Grade 3 or higher toxicities were rare (<10% incidence) and mostly haematologic: anaemia or thrombocytopenia. Both studies confirmed that BRCA1/2 mutational status serves as predictive markers for PARPi.

### PARPi combination trials

Preclinical studies indicated enhanced cytotoxic effect from the addition of PARPi to platinum analogues in HR-defective cancer. Homologous recombination defects are commonly seen in triple-negative breast cancer and include BRCA1 methylation, overexpression of de-regulators including ID4 and HMG as well as aberrations of MRE11, ATM and PALB2 (Alli *et al*, 2009; Alexander *et al*, 2010). Therefore, these cases are appropriate targets for PARP inhibition. Iniparib was recently combined with gemcitabine and carboplatin in a randomised phase II trial in 123 patients with triple-negative breast cancer including those who had received prior chemotherapy for metastatic disease (O’Shaughnessy *et al*, 2011). The primary study end point was disease control (partial response+stability) and iniparib increased disease control rate (from 34 to 56%), response rate (from 32 to 52%), progression-free survival (from 3.6 to 5.9 months) and overall survival (from 7.7 to 12.3 months) without increasing toxicity. These promising results in the phase II setting led to the first PARPi phase III study that enrolled over 500 patients. However, this phase III study did not meet the prespecified criteria for significance for co-primary end points of overall survival and progression-free survival, although patients who had received 1–2 prior chemotherapy regimens appeared to benefit (Guha, 2011). The negative results of this phase III study are clearly a setback in this field. However, since the mechanism of action of iniparib is not clearly understood, caution must be exercised in attributing these results as a possible ‘class effect’. This was illustrated by earlier reports of GAPDH inhibition (Bauer *et al*, 2002) and more recently in PD studies of various PARPi where a dose- and time-dependent inhibition of PARP formation was observed with veliparib, olaparib and MK-4827 but not with iniparib (Ji *et al*, 2011). In this study, *γ*-H2AX induction occurred with all agents, including iniparib, suggesting other mechanisms of action for iniparib besides PARP inhibition. Furthermore, there were important differences between the above phase II and III iniparib studies. The phase II was an open-label study with the primary end point of clinical benefit whereas the phase III was a placebo-controlled, blinded study with survival as the primary end point. This study did not check for BRCA1/2 mutation status; only 20% of triple-negative breast cancers exhibit these mutations (Gonzalez-Angulo *et al*, 2011). Finally, this trial included gemcitabine as a chemotherapeutic agent, which does not exhibit synergistic anti-tumour activity with PARPi.

### Toxicity concerns

As discussed above, myelosuppression is being increasingly recognised in PARPi combination trials with chemotherapy, particularly where PARPi are being dosed continuously rather than intermittently. The mechanism is unclear at this time but preclinical data suggest that much higher doses of PARPi are tolerated as a single agent compared to in combination with cytotoxics, for example in mice the MTD of AG014699 in combination with temozolomide is 1 mg kg^–1^ but as a single agent 25 mg kg^–1^ is completely non-toxic (Thomas *et al*, 2007; Drew *et al*, 2010). PARPi may result in long-term toxicities from prolonged DNA repair inhibition that must be cautiously evaluated in clinical trials. Prolonged DNA repair inhibition may paradoxically result in secondary cancers. Disruption of PARP-1 caused a high incidence (49%) of aggressive brain tumours in p53 null mice, with typical features of human cerebellar medulloblastomas, thus implicating PARP-1 in tumour suppression (Tong *et al*, 2003; Rouleau *et al*, 2010). PARP-1 knockout mouse models were also susceptible to obesity and insulin resistance (Devalaraja-Narashimha and Padanilam, 2010). However, there is wide inter-individual variability of PARP activity in humans, thus potentially limiting toxicity to subpopulations only (Zaremba *et al*, 2011).

### Biomarkers with predictive value for PARP inhibition

The identification of HR defects in cancers (beyond BRCA1/2 mutations) may potentially indicate sensitivity of PARPi as discussed above. A recent study identified a BRCA-like 60-gene signature profile in familial and sporadic ovarian cancers (Konstantinopoulos *et al*, 2010). The predictive accuracy of this gene signature was validated initially in 10 tumour biopsies from 6 patients with germline BRCA1/2 mutations and in 70 patients with sporadic ovarian cancer and significant correlation was noted with platinum sensitivity and clinical parameters including survival. On a multivariate analysis, which included the *BRCA-*ness profile, age, stage, grade, histology and debulking status, the profile maintained an independent association with disease-free and overall survival. An alternative approach is to perform assays of HR function. DNA damage-induced RAD51 nuclear focus formation is the hallmark of HR (with no increase in foci after DNA damage in HR-defective cells) and thus RAD51 nuclear foci have been used as surrogate markers for HR. Mukhopadhyay *et al* (2010) investigated RAD51 foci formation in 25 primary ovarian cancer cultures; failure to form foci correlated with *ex vivo* sensitivity to AG014699 with a negative predictive value of 100% and positive predictive value of 93%. In this study, a 50–60% incidence of HR deficiency in sporadic ovarian cancers was reported. Similar, smaller studies in core biopsies from breast cancers and AML show that DNA damage-induced RAD51 foci can be detected in different tumour types (Gaymes *et al*, 2009; Willers *et al*, 2009). Another study investigated RAD51 nuclear foci in formalin-fixed, paraffin-embedded samples of breast cancer surgically excised after neoadjuvant anthracycline therapy. Their results showed that defective HR, as indicated by low RAD51 foci, may predict response (Graeser *et al*, 2010). These RAD51 foci assays may indicate potential responsiveness to treatment with PARPi but the tissue requirement can be problematic as the cells are required to be in S-phase for an accurate assessment. Functional loss of BRCA1/2 and biomarkers including PALB2, FANCF, RAD54, PTEN, EMSY, XRCC2, XRCC3 in tumour biopsy specimens could potentially also have predictive value for PARP inhibition and need to be prospectively investigated in clinical trials. Clearly, none of these assays are candidates for routine clinical practice and it will be necessary to develop simple, cost-effective methods to identify HR defects for effective and appropriate patient selection for PARPi therapy.

### Considerations for future clinical trial designs for PARPi

It is clear from the preclinical evidence and emerging clinical evidence that a number of considerations need to be taken into account when designing PARPi clinical trials. These are different depending on whether the PARPi is to be used as a single agent or in combination, and whether to be given to ‘all comers’ or restricted to those patients with HR-defective tumours. When targeting HR-defective tumours these need to be identified reliably. To date, the most robust method seems to be RAD51 focus formation but these assays are not trivial to perform nor widely applicable in solid tumours. Haematological malignancies may be easier to stratify. Clearly, assuming all triple-negative breast cancers to be HR defective is not supported by the evidence (Graeser *et al*, 2010) and so these may not be the ideal cancer population. However, patients with high-grade serous ovarian cancer may be a more promising target population as the evidence suggests at least 50% have HR defects (Mukhopadhyay *et al*, 2010).

From the preclinical data it would appear that higher doses and prolonged, continuous single-agent PARPi therapy is needed for optimum effect. Presumably, this is because anti-tumour activity is dependent on maximum inhibition of the low-level endogenous damage in S-phase cells. In contrast, in combination studies, less profound PARP inhibition is needed to enhance cytotoxic agent-induced DNA breakage and anti-tumour activity (Calabrese *et al*, 2003, 2004; Daniel *et al*, 2009). In combination doses of both agents must be carefully titrated to achieve a therapeutic effect without markedly increasing toxicity. Much lower doses and shorter durations of PARPi therapy are likely to be optimum in combination studies (Calabrese *et al*, 2003). Based on the preclinical evidence discussed above, combinations with TMZ or DTIC, topoisomerase I poisons and ionising radiation are the only ones likely to be effective in ‘all comers’ and combinations with cis or carboplatin are likely to be effective for HR-defective tumours. Any enhancement of anti-metabolites, topoisomerase II poisons, bifunctional alkylating agents or anti-tubulin agents will be dependent on the potential vasoactivity of PARPi, which has only so far been reported preclinically for two inhibitors (Ali *et al*, 2009).

## Conclusions and future directions

Over 30 years of research since 3-AB was first shown to inhibit DNA repair and increase alkylating agent cytotoxicity has culminated in the clinical investigation of at least nine PARPi. The preclinical data show robust sensitisation of TMZ, topoisomerase I poisons and irradiation as well as synthetic lethality in HR-defective cancer. The data beginning to emerge from the clinical trials largely bear out the preclinical data. It is clear from the preclinical data that much higher doses of PARPi are tolerated as a single agent than in combinations with cytotoxic agents. This observation may underlie the toxicities observed in the PARPi combination trials using the safe PARPi dose that had been determined in single-agent studies. Conversely, where the safe PARPi dose and schedule has been determined in combination with a cytotoxic, it may be insufficient to have a therapeutic effect as a single agent. It is evident that single-agent PARPi have broader application than initially supposed as HR defects are far commoner than BRCA1/2 mutations. Assessment of HR status by looking at markers of HR function is reliable but not trivial, the challenge is now to develop a simple method to identify these HR-defective tumours. A number of options are under investigation such as sequencing and expression analysis of key genes, determination of an HR defect-specific gene signature and IHC for key proteins. A functional assay is probably going to be needed as a ‘benchmark’ by which to validate these alternative assays. Haematological malignancies will be the easiest to investigate in the first instance due to the more readily accessible tumour material. The assay that is finally adopted will be key to the success of PARPi and the continued move to personalised medicine, where the molecular pharmacology of the tumour, rather than its tissue of origin dictates the appropriate therapy.

## Figures and Tables

**Table 1:**
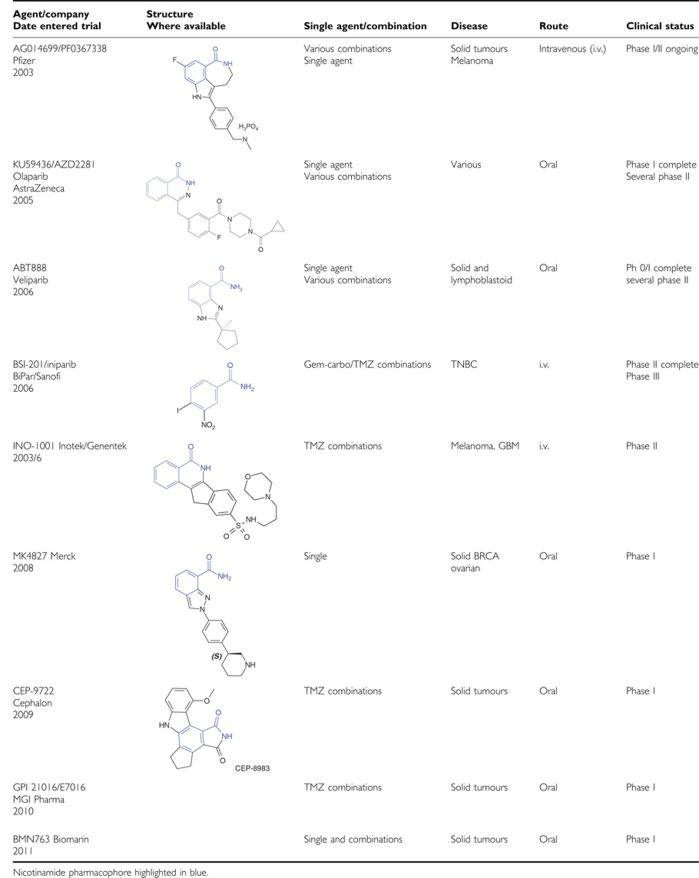
PARP inhibitors in clinical development
